# Adenocarcinoma arising from a foregut cyst of the diaphragm: importance of multimodality treatment: a case report

**DOI:** 10.1186/s12893-020-01005-1

**Published:** 2020-12-14

**Authors:** Jozsef Furak, Anna Rieth, Aurel Ottlakan, Tibor Nemeth, Laszlo Torday, Laszlo Tiszlavicz, Gyorgy Lazar

**Affiliations:** 1 Department of Surgery, Faculty of Medicine, University of Szeged, Semmelweis str. 8, Szeged, 6725 Hungary; 2 Department of Pediatric Surgery, Faculty of Medicine, University of Szeged, Koranyi alley 14-15, Szeged, 6725 Hungary; 3 Department of Oncology, Faculty of Medicine, University of Szeged, Korányi alley 12, Szeged, 6720 Hungary; 4 Department of Pathology, Faculty of Medicine, University of Szeged, Allomas str. 2, Szeged, 6725 Hungary

**Keywords:** Foregut cyst, Diaphragm, Adenocarcinoma, Multivisceral resection, Multimodality treatment

## Abstract

**Background:**

Benign foregut cysts usually develop in the thorax most of all in the mediastinum. Rare cases involving various abdominal organs, such as liver, stomach or pancreas have been previously published, mostly occurring in the retroperitoneum.

**Case presentation:**

We herein present an adenocarcinoma of a foregut cyst involving the left side of the diaphragm, left lower lobe of the lung, and left lobe of the liver, successfully removed through multivisceral resection. In between drug holidays, postoperative oncological treatment has been ongoing for nearly 4 years. In terms of chemotherapy, FOLFOX 4 regime, capacitabine monotherapy and later on next generation sequencing has been attempted, although the patient refused the later treatment option. Despite multimodality (combined surgical and oncological) treatment, local- and later on loco-regional recurrence has been detected on follow-up staging, influencing further chemotherapy regime. Taking both the fairly unknown type of the tumor and uncertain response rate to oncological therapy into account, prolonged tumor pace with fairly stable general patient state was reached throughout the course of the disease.

**Conclusion:**

Through surgical tumor resection, and postoperative chemotherapy the patient managed to maintain an acceptable quality of life without major symptoms during ongoing treatment. During our own case, with multiple organ involvement, multivisceral resection, with multimodality treatment had considerable effect in prolonging the lifespan of the patient.

## Background

Until recently, about 200 primary diaphragm tumors have been reported worldwide [1]. Primary benign lesions include mesothlial cysts, mostly occurring in children, and tumors arising from bronchogenic cysts [2]. The most frequent benign tumors of the diaphragm include lipomas, with the first case reported in 1886, by Clark [3]. However, benign lesions, such as lipomas may in some percentage transform into liposarcomas, thus resection of these seemingly harmless lesions is in most cases mandatory [4]. Malignant diaphragm lesions include rhabdomyosarcomas, usually with poor prognosis, although patients often benefit from the combination of surgical resection, chemotherapy and postoperative irradiation [5]. Leiomyosarcomas occur relatively rare at the site of the diaphragm, leaving only surgical resection as a feasible treatment option [6]. Secondary malignant lesions of the diaphragm often occur due to neighbouring malignant tumors, most frequently lung cancer, mesothelioma, or thymomas [7]. Adenocarcinoma of a foregut cyst (AFC) to our knowledge has only scarcely been described in literature. Tumors, such as AFC, require special treatment, and handling, due to the lack of information on tumor behaviour and available treatment options. Our own case not only deals with a previously undescribed malignancy, but due to multivisceral involvement, also resulted in personalized, multimodality, combined oncological and surgical treatment (multivisceral resection), leading to prolonged lifespan and acceptable quality of life for the patient.

## Case presentation

**Fig. 1:**
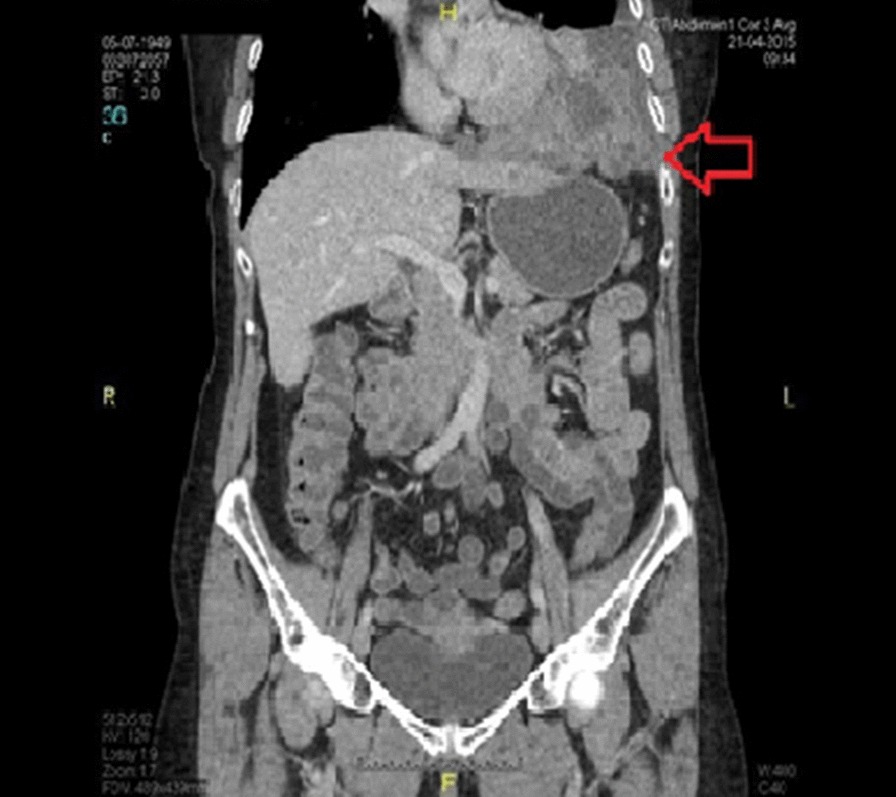
Preoperative computed tomography (CT) showing the lesion on the left diaphragm (red arrow)

**Fig. 2:**
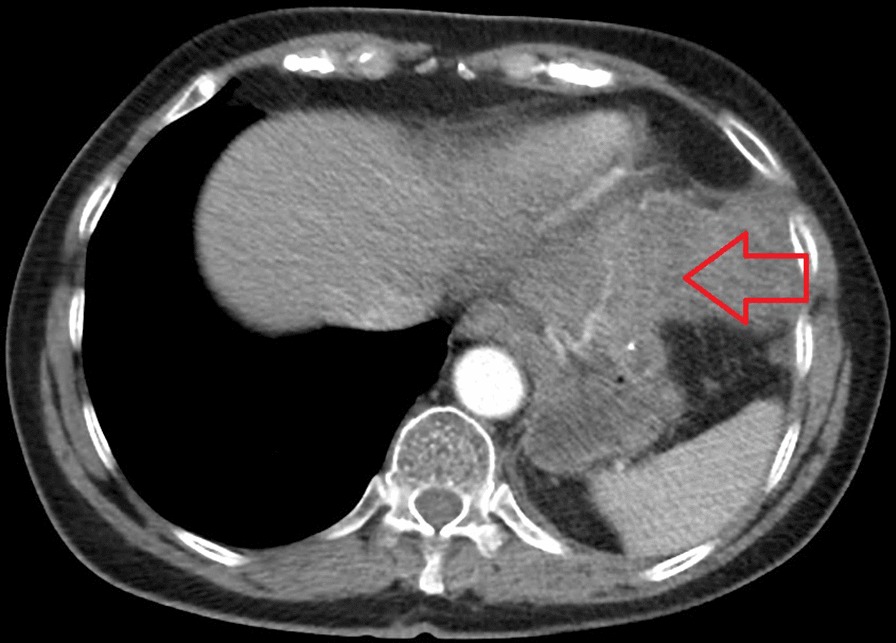
Preoperative cross-sectional CT image of the tumor (red arrow)

A 66-year-old woman presented at our Department with chest-, and abdominal pain. During work-up, contrast enhanced chest- and abdominal computed tomography (CT) verified a large mass (10 × 6 × 5.5 cm) located in the left diaphragm, suggesting infiltration of the fundus of the stomach and the left lobe of the liver. An exophytic mass measuring 1.5 cm in diameter was seen at the posterior wall of the stomach, with low amount of calcification. A 13 mm large lymphnode, on the left side, posterior to the crus of the diaphragm was visible, otherwise there were no pathologically enlarged lymphnodes in the abdomen, or the pelvis (Figs. 1, 2). The patient had no history of malignancies, and at initial presentation no distant metastases were confirmed on imaging. Left sided atelectasis and pleural fluid were also described on chest CT. Gastroscopy was negative. Ultrasound guided fine needle biopsy of the mass suggested a malignancy consisting of epithelial cells. On the basis of these findings, primary surgical approach was indicated in concordance with our tumor board.

After video-assisted thoracic surgery (VATS) exploration excluded pleural dissemination, a left sided posterolateral thoracotomy was performed in the 9th intercostal space. The tumor was located in the left diaphragm with infiltration of the left lower lobe of the lung and left lobe of the liver. Regarding pulmonary involvment, the lesion was situated at the base of the left lung, in close proximity to the pericardium, with medial border reaching the diaphragmatic crura and lateral border on the thoracic wall. There were no potential cysts, or pseudocysts identified during the surgical procedure.

**Fig. 3:**
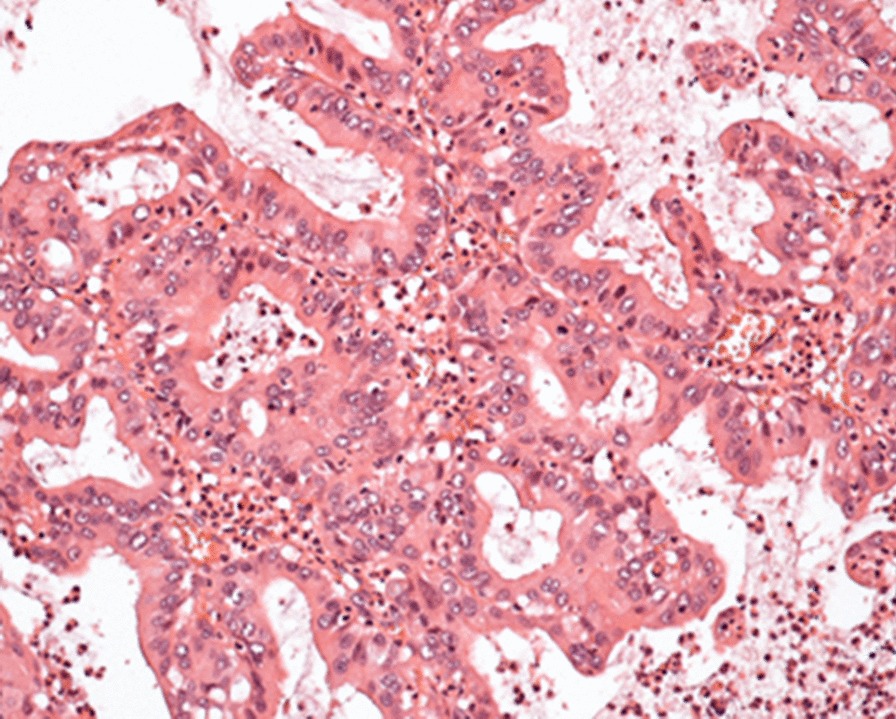
Typical acino-papillary pattern of well-moderately differentiated adenocarcinoma with reactive granulocytes (Hematoxylin–eosin staining, 10×)

**Fig. 4:**
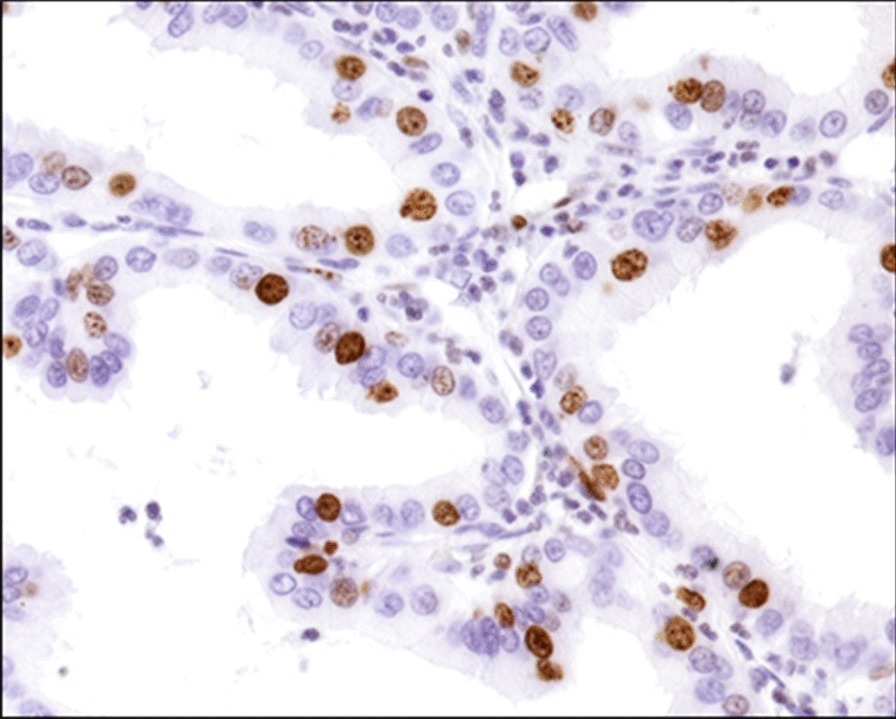
The nuclear positivity of proliferation marker (braunish) affecting 25% of tumor cells (Immunohistochemistry, Ki-67, 40× )

Multivisceral resection was carried out, involving resection- and reconstruction of the left diaphragm (Gore-tex® Dualmesh®), wedge resection of the left lower lung lobe and resection of the left liver lobe. The tumor did not involve the stomach. Postoperative period was uneventful, and the patient was discharged after 7 days. Histology revealed the following findings: (1) resected lung specimen (wedge-resection): BOOP (Bronchiolitis Obliterans Organizing Pneumonia)-like picture: intraalveolar, organizing fibrotic tissue, with significant interstitial chronic cell inflammation. Secondary vascular pulmonary hypertension, without presence of malignancy in the specimen. (2) Peritoneum: fibrotic pseudocysts without inner epithelial lining, PAS-AK dying suggested the presence of mucus, rich in acidic mucosacharids. There was no detectable vital tumor tissue in the sample. The presence of necrotic metastases was suggested. (3) Resected diaphragm: Previously mentioned pseudocysts detected besides striated muscle-, and liver tissue, with extended necrosis, accumulated neutrophil granulocyts, and focal vital tumor tissue. A well-moderately differentiated adenocarcinoma with a papillary pattern was confirmed, with high amount of polysacharids and mucin. Immune-phenotyping showed focal positivity for CK7, MucAc (3 +), Ki67 (3 +), p53 do-7 clone (3 +) and CDX2 and negative reaction for TTF1 (Transcription Termination Factor 1), CK20, Napsin A, Thyreoglobulin, and Surfactant protein immune acid (Figs. 3, 4). On the basis of the above findings, a well-differentiated, low-grade adenocarcinoma arising from a foregut cyst was confirmed. Surgical resection margins proved to be clear, resulting in complete, R0 resection.

**Fig. 5:**
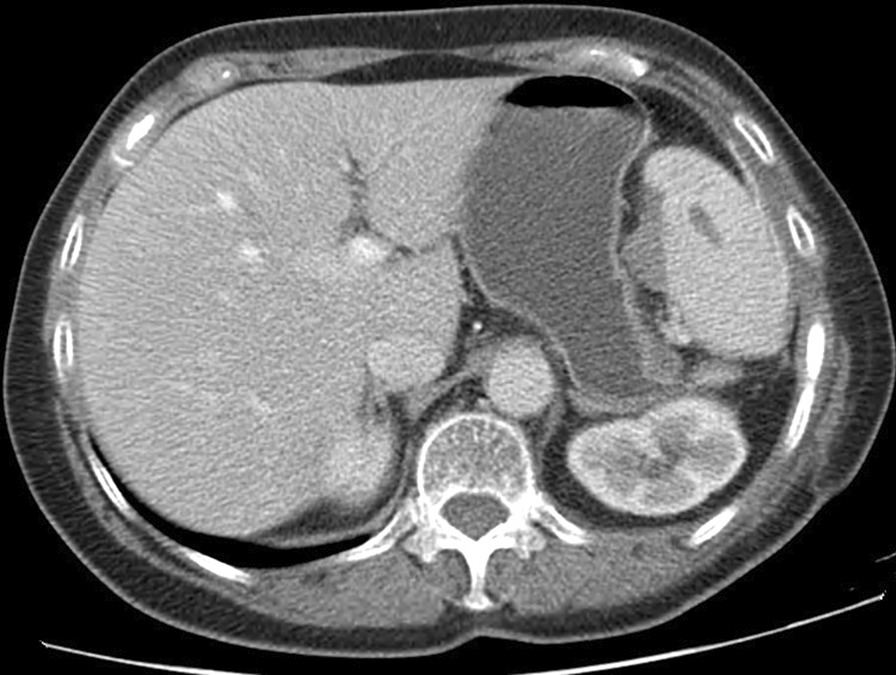
Postoperative CT, 1 month after surgery, showing tumor-free state

After the procedure, no postoperative chemotherapy was initiated due to both clear surgical resection margins and strong patient preference. Follow-up CT one month after surgery showed a tumor-free state (Fig. 5).

**Fig. 6:**
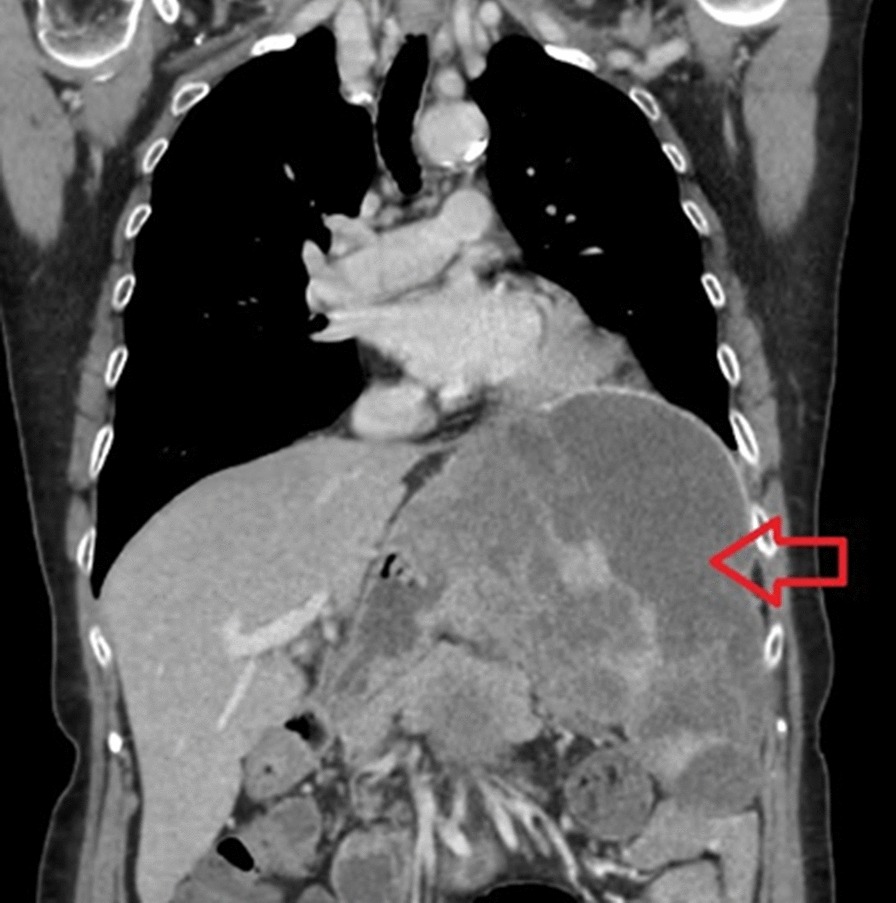
Postoperative CT showing loco-regional failure at the site of the spleen (red arrow)

**Fig. 7:**
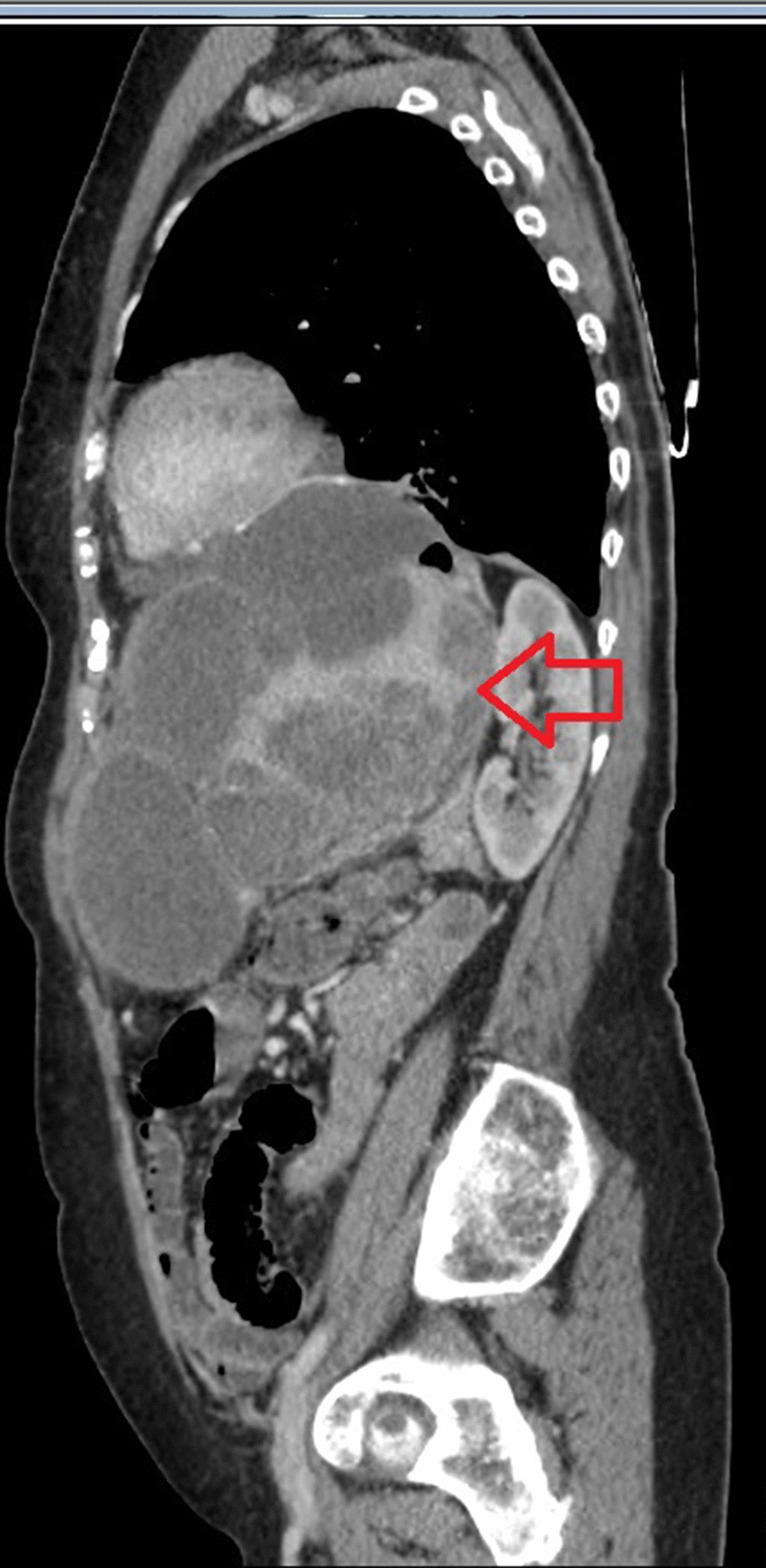
CT-sagittal view of recurrent tumor at the site of the spleen, 3 year after surgery (red arrow)

**Fig. 8:**
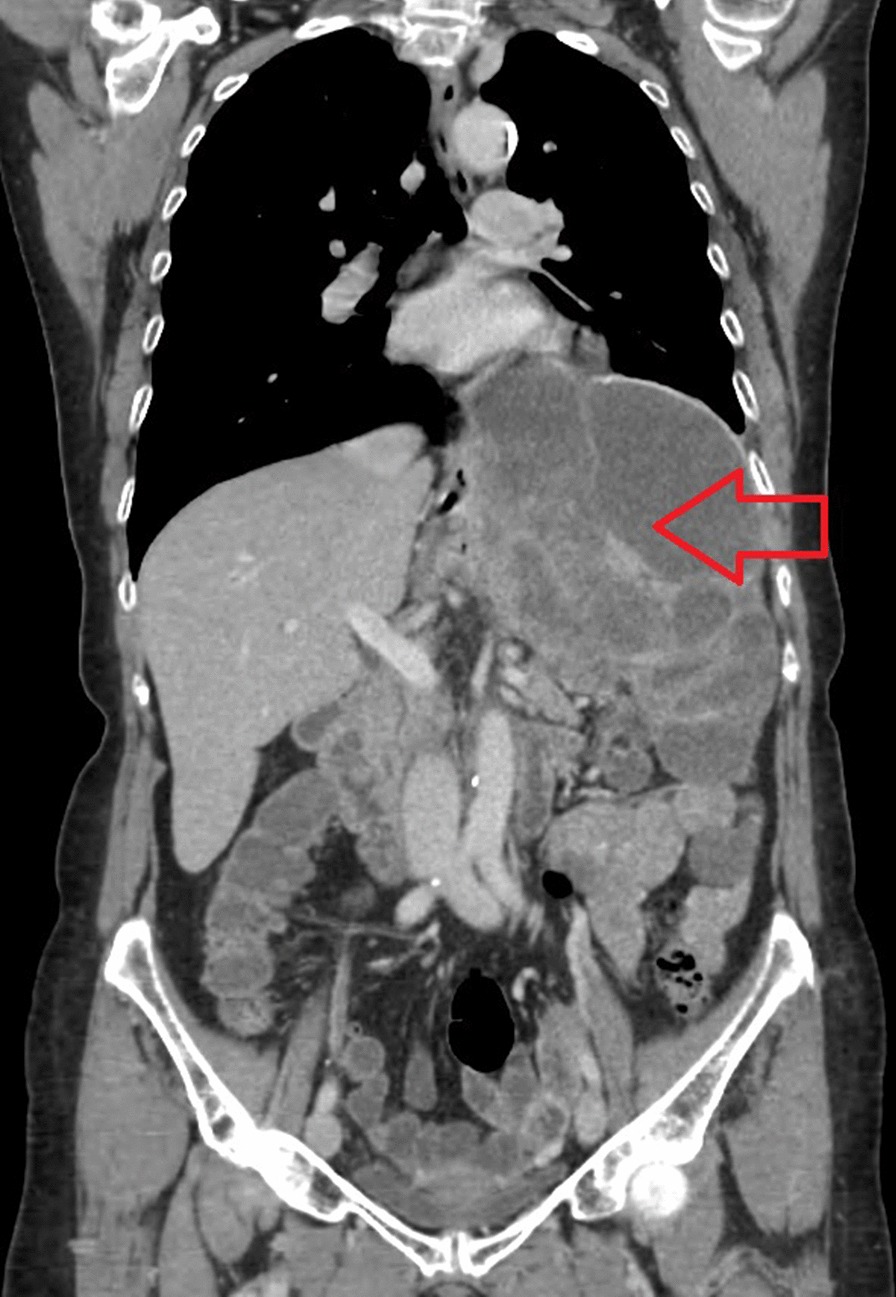
CT-frontal view of recurrent tumor at the site of the spleen, 3 years after surgery (red arrow)

**Fig. 9:**
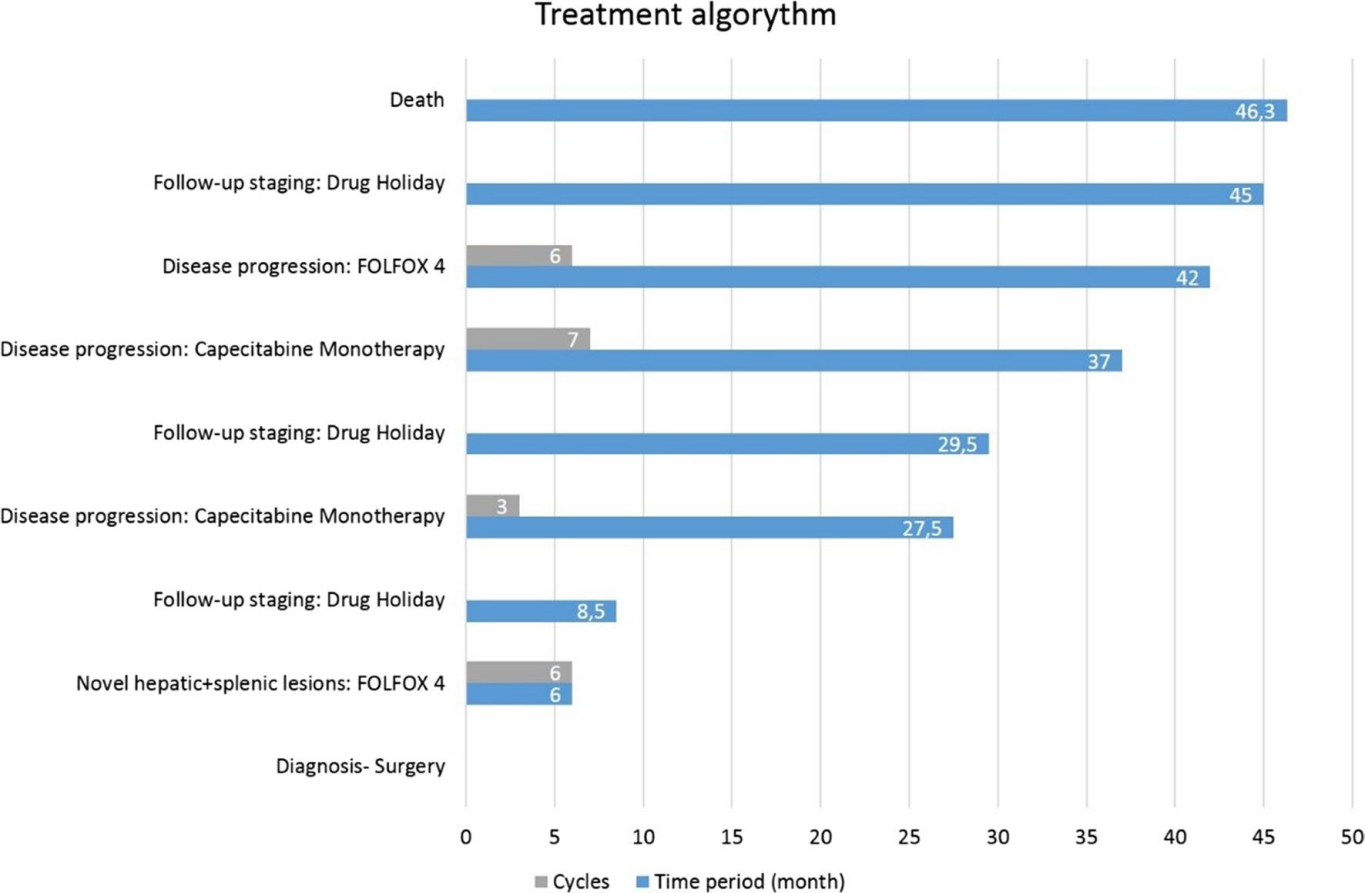
Treatment algorithm of primary adenocarcinoma of the diaphragm

Six months after surgery, during regular follow-up, novel hepatic and splenic lesions have been detected on abdominal CT. After a further two months, additional disease progression was confirmed involving both hepatic and splenic metastases (Fig. 6). PET-CT (positron emission tomography-computed tomography) showed distant metastases within the left liver lobe and left supraclavicular lymph nodes. A splenic lesion has been described involving the gastric wall at the lesser and greater curvature with neighbouring lymphnode enlargement. As the next treatment approach, palliative FOLFOX4 chemotherapy has been initiated. After 5 cycles of treatment (2.5 months) due to the lack of any physical symptoms and stable disease confirmed on follow-up CT, the patient refused further oncological treatment, thus a drug holiday was initiated with regular follow-ups and tumor staging. After 19 months without any chemotherapy (drug holiday), the patient started complaining of worsening abdominal pain and cramps. Urgent CT confirmed disease progression, hence capecitabine monotherapy was started. After initiation of this treatment, symptoms rapidly improved. Three cycles (2 months) were delivered, and new imaging (CT) was carried out, confirming further, slight increase concerning the splenic lesion. We suggested continuing chemotherapy with an enhanced regimen, the patient however refused to continue treatment on the grounds of lacking symptoms. After a 7.5 month drug free period, follow up CT confirmed considerable progression of splenic metastases. The patient expressed clear preference for oral therapy, thus capecitabine was reinitiated (Figs. 7, 8). After finishing 7 new cycles (5 months), progression was once again detected on imaging. Afterwards, the patient was succesfully convinced to accept intravenous chemotherapy, so treatment regimen could be enhanced to FOLFOX4. With this regimen, stable disease was achieved after 6 cycles (3 months) although treatment was once again discontinued due to patient request. Considering the fact that on one occasion capecitabine improved tumor related symptoms and both capecitabine and FOLFOX4 stopped further disease progression, both regimens seemed to be effective in the management of the disease. Throughout the oncological management, the best presumed therapy was taken into consideration, in concordance with patient preference, and taking the course of the disease with best achievable response into account in maintaining a relatively stable patient condition and tumor stage. Facing a rare occurring tumor without established oncological treatment options, and patient rejection of cytotoxic chemotherapy, in order to identify potentially targetable driver mutations, next generation seqencing (NGS) was requested as a subsequent step. The FDA (Food and Drug Administration) approved FMI CDx (FoundationOne® Companion Diagnostic) assay was applied which analysed 315 genes as well as introns of 28 genes involved in rearrangements. The following relevant genomic alterations were identified: ATM E522fs*43 mutation, KRAS Q61K mutation, PBRM1 rearrangement intron 10. The loss of functional ATM results in a defective DNA damage response and homologous recombination-mediated DNA repair, and may predict sensitivity to PARP inhibitors such as olaparib, rucaparib, and niraparib. Preclinical evidence suggests that KRAS activation may predict sensitivity to MEK inhibitors, such as trametinib and cobimetinib. There are no targeted therapies available to address genomic alterations in PBRM1. On the basis of these findings and with confirmed disease progression, the application of one of the PARP inhibitors was intended, however, despite detailed description of potential health benefits, the patient refused to accept recommended treatment. After 39 days the patient was eventually admitted to the 1st Department of Medicine with acute hematemesis. Controll gastroscopy confirmed a large tumorous infiltration inside the fornix of the stomach without acute bleeding at the time of intervention. However one day after admission, general patient condition showed rapid progression with cardiac- and respiratory insufficiency and the patient passed away on the following day [nearly 4 years (1389 days) after surgery] (Fig. 9).

## Discussion and conclusions

Foregut cysts (FC) are congenital developmental abnormalities of the foregut occurring during embryogenesis. Although FCs are most frequently found in the mediastinum, due to their assumed origin from bronchial branches, in some cases they can also descend to the abdominal cavity, leading to the development of an abdominal FC. Malignant transformation of FCs are extremely rare, however cases have been reported on transformed cysts in the retroperitoneum and in organs of the abdominal cavity [8].

Curative treatment of such multiple organ involving malignancies may well be surgery, or palliative treatment (bypass, palliative chemotherapy) at the most in unresectable cases. Our case involved multiple resection of intra-abdominal organs (diaphragm, lung, liver), followed by adjuvant chemotherapy, with anticipated prolonged survival of the patient, which has been confirmed during follow-up (despite regional recurrence), since the patient maintained acceptable quality of life throughout a relatively long time period (1389 days after surgery). In cases such as ours, the pivotal role of multimodality treatment should be highly emphasized. In cases such as AFC, the lack of previous treatment experience and unknown response to oncological treatment may hamper successful therapy. The application of personalized treatment strategy and targeted therapy could lead to improved quality of life and survival. It is well described that cancer development is in close connection with the genome [9]. The use of various sequencing modalities has enabled us to identify genetic aberrations in numerous tumors. Tumor heterogeneity plays a pivotal role in applying targeted therapies, due to the fact that it is not only found among different patients, but also within the same patient at various sites (intrapatient heterogeneity), or even within the same tumor, at different locations (intratumor heterogeneity) [9]. Next generation sequencing results in a highly specific diagnosis based on genotype. During NGS, cancer specimens are analysed for fundamental alterations, such as base substitutions, insertions and deletions, copy number alteration, and rearrangements [10]. According to a prospective, multi-center study, among 86 cancer patients (colorectal, breast, ovarian) undergoing genetic profiling, a molecular target was identified in 98%. Eighteen patients (27%), received genomically guided treatment (GGT) and successfully reached progression-free survival, thus GGT was found beneficial and feasible in these cases [11]. In a study conducted by Hasselgren et al., involving 42 patients undergoing multivisceral resections, no significant correlation between postopertaive morbidity, number of resected organs and number of anastomoses was noted, however a tendency in postoperative complications became apparent in cases where more than 4 organs were resected [12]. In similar operable cases, even with potentially increased morbidity, multivisceral resection seems to be the only feasible solution [12]. Treatment options of malignant lesions arising from FCs have previously been described in literature, although mostly with palliative management and the lack of appropriate follow-up, or survival data [13]. Multivisceral resections involving hepatectomy, or pancreatectomy combined with at least one other organ resection carry a 7% postoperative mortality rate with 59% complication rate [12], however postoperative recovery was uneventful in our case, without any perioperative morbidity. Nonetheless, according to a retrospective analysis by McKay, et al. on the postoperative outcomes of multivisceral resections, disease free survival after combined hepatic and pancreatic resections was 10 months and after multiorgan resections involving hepatic-, or pancreatic resection, 3 year overall survival was 79% [12]. Based on our own case,—despite loco-regional failure at 6 months-, oncological treatment through postoperative chemotherapy successfully resulted in an acceptable quality of life, in spite of distant metastases occuring later on. The nearly 4 year (1389 days) survival rate, along with the previously mentioned tumor recurrence shows, that due to its low grade malignant features, the pace of tumor advancement was still moderate, resulting in a prolonged course of the disease. Taking aspects of tumor development into consideration, multimodality treatment through aggressive surgical resection and postoperative chemotherapy was deemed a relatively successful approach.

### Limitations

There are some limitations to the above discussed case.

1. Our case included a little known, highly complex malignant lesion, without previous data of definitive pre-, or postoperative oncological treatment. After histology confirmed tumor origin, postoperative treatment was initiated based on similar foregut derived malignancies, since no previous adjuvant treatment plan served as a model, on which our own treatment strategy could have been constructed.
2. Since scarce information on the malignant potential of the lesion, it was extremely difficult to predict treatment response and outcome.
3. Authors agree on the fact, that based on a single case, no far-reaching conclusions can be drawn from the above mentioned condition in terms of treatment algorithm and life expectancy. Careful patient selection and thorough preoperative work-up certainly may facilitate successful surgical/oncological therapy.
4. With the detailed discussion and treatment recommendation through the case, the authors hope to have made an effort to better understand foregut derived malignancies and added valuable information on overall treatment planning, contributing to improved survival rates.

## Data Availability

The datasets used and/or analysed during the current study are available from the corresponding author on reasonable request. All data generated or analysed during this study are included in this published article [and its supplementary information files].

## Abbreviations

CT: Computed tomography
FC: Foregut cyst
FDA: Food and Drug Administration
GGT: Genomically guided treatment
NGS: Next generation sequencing
PAD: Primary adenocarcinoma of the diaphragm
PET: Positron emission tomography
TTF1: Transcription termination factor 1
VATS: Video-assisted thoracoscopic surgery
